# Smoking Uptake Among Adolescents in Social Housing Australia

**DOI:** 10.1093/ntr/ntae207

**Published:** 2024-09-05

**Authors:** Ankur Singh, Erika Martino, Adelle Mansour, Rebecca Bentley

**Affiliations:** Centre for Epidemiology and Biostatistics, Melbourne School of Population and Global Health, University of Melbourne, Melbourne, Victoria, Australia; Melbourne Dental School, University of Melbourne, Melbourne, Victoria, Australia; Centre for Health Policy, Melbourne School of Population and Global Health, University of Melbourne, Melbourne, Victoria, Australia; Centre for Health Policy, Melbourne School of Population and Global Health, University of Melbourne, Melbourne, Victoria, Australia; Centre for Health Policy, Melbourne School of Population and Global Health, University of Melbourne, Melbourne, Victoria, Australia

## Abstract

**Introduction:**

Australia’s limited social housing has created geographically concentrated locales of poverty with high smoking rates. The impact of social housing on smoking initiation among adolescent residents is unknown, despite adolescence being a critical period for smoking prevention. We examine the relationship between social housing residency and smoking initiation among adolescents to quantify the likelihood of smoking uptake among social housing residents compared to a similar cohort in other tenures, accounting for socioeconomic factors and household exposure to smoking.

**Methods:**

We analyzed data on 15- to 18-year-old adolescents (*n* = 3132) from the Household, Income and Labour Dynamics in Australia survey (2001–2019). We applied inverse probability treatment weights to maximize exchangeability between social housing tenants and their counterparts in other tenures. We quantified the risk of smoking 5 years after exposure measurement among those in social housing on both an absolute and relative scale. Baseline covariates included household income, age at study entry, sex, family type, smoking at baseline, highest household education, and household exposure to smoking.

**Results:**

Adolescent residents in social housing had a 17% greater risk of smoking 5 years after baseline measurement than their counterparts in all other tenures (Average Treatment Effect (ATE): 0.165, 95% confidence interval [CI] = 0.02 to 0.31). On the relative scale, those in social housing had 1.80 times (95% CI = 0.95 to 2.66) higher risk of being a smoker than those in other tenures.

**Conclusions:**

Adolescents residing in social housing have a higher risk of becoming smokers as young adults than their counterparts in other tenures, irrespective of smoking exposure in their own homes.

**Implications:**

This study investigates the impact of social housing on smoking initiation among adolescents, revealing that those residing in social housing have a higher risk of becoming smokers in young adulthood, independent of smoking exposure at home. The research highlights the contribution of social housing to ongoing disparities in smoking rates in Australia and emphasizes the need to further understand and review social housing provision from the perspective of its consequences on health. Moreover, the results advocate for comprehensive policies that extend beyond individualized harm reduction strategies to promote social inclusion and address health inequalities associated with smoking in adolescents.

## Introduction

Tobacco smoking is the leading contributor to the total global disease burden.^1^ In high-income nations, smoking rates are falling; however, they remain socially patterned with people who experience social disadvantage more likely to smoke.^2,3^ While much literature on smoking prevention has focused on intervening on individuals and their lifestyle choices, there is a growing recognition that social context and residential environments are strong determinants of smoking.^4–6^ Social contexts related to smoking are an important dimension of power relations whereby social inequalities define the access to material resources, human resources, and control of ideas.^7^ The social and geographic patterning of smoking parallels the effects of other processes of marginalization and social disadvantage.^7^ Persistently higher smoking rates have been reported in the rental and public housing sectors.^8–14^ While this is correlated with socioeconomic stratification by tenure, the nature of our housing system as a determinant of smoking behavior should also be considered as a possible intervention point if we are to reduce social inequalities in smoking patterns.

When comparing smoking outcomes by tenure, the highest rates of smoking and lowest cessation rates are observed among social housing tenants across many countries.^12,15^ Explanations that have been given for this include increased exposure to psychosocial stressors experienced by social housing tenants (likely exacerbated by being located in low socioeconomic neighborhoods)^4,16^; greater prevalence of depression and poorer mental health among social housing tenants^17^; and the increased visibility of smoking and smoking products in people’s homes and local neighborhood. These factors are thought to converge to increase smoking uptake and foil campaigns to prevent smoking by reducing advertising resistance and deterring antismoking attitudes and refusal to smoke.^18^ Less consideration has been given to examining how the provision and governance of social housing contribute to social conditions that foster higher rates of tobacco use. The high rates of smoking in social housing in adults could lead to easier access of cigarettes for adolescents, and also increase perceived norms related to cigarette smoking. Given this, evidence of the potential importance of residential context can be found in the comparison of smoking rates between residents of the private and public rental sectors.

One way to disentangle the contribution of a residential context such as social housing tenure to smoking uptake is to compare matched cohorts of young people across different tenure types over the same period. Given that most people take up smoking in adolescence and early adulthood^19^ when early experimentation with tobacco is a predictor of use in adulthood,^20,21^ it makes sense to focus on young cohorts in particular. By creating cohorts of young people who are similar in terms of their sociodemographic profile, we can disentangle the extent to which smoking rates are related to specific residential contexts and how they track over time to create disparities by tenure. In this study, we use annually collected longitudinal data to quantify changes in cigarette smoking across time and by tenure for 15- to 18-year-old adolescents to examine if countrywide tobacco control measures have similar effects on comparable cohorts. Second, we examine the association between living in social housing and smoking uptake in adolescents compared to smoking initiation rates of young people in other tenures. Specifically, we consider whether young people between 15 and 18 years of age who reside in social housing are at greater risk of becoming smokers after 5 years when compared to their counterparts in other tenures—as a driver of widely described tenure-based smoking disparities.

## Methods

### Data Source and Study Population

Our study population was sourced from the longitudinal Household, Income and Labour Dynamics in Australia (HILDA) survey, a nationally representative sample of Australian households with a specific focus on family and household formation, income, and work.^22^ Commencing in 2001, the original sample included over 13 969 participants from over 7682 households at baseline. The sample has been gradually extended to include any new household members. In 2011, a top-up sample of 2153 households was added to maintain representativeness.^23^ Analysis was conducted on all 19 waves of data from 2001 until 2019. Data were collected via interviews and self-completion questionnaires. The analytical sample consisted of adolescents aged 15–18 years, who were followed for a subsequent 5 years (20–23 years old).

### Study Measures

#### Outcome

Current smoking among adolescents 5 years from the baseline measure of their housing and smoking status was selected as the outcome measure. At each annual wave between 2002 and 2019, participants were asked if they smoke cigarettes or other tobacco products. The responses included “No, I have never smoked,” “No, I no longer smoke,” “Yes, I smoke daily,” “Yes, I smoke at least weekly (but not daily),” and “Yes, I smoke less often than weekly.” We categorized those reporting “Yes, I smoke daily,” “Yes, I smoke at least weekly (but not daily),” and “Yes, I smoke less often than weekly” as current smokers and “No, I have never smoked” and “No, I no longer smoke” as nonsmokers (reference category).

#### Exposure

Housing tenure was selected as the exposure measure, categorized as “outright owners,” “mortgage,” “private renter,” “public renter,” and “other.” From these groups, we generated a binary measure of social housing exposure (public renter) and nonsocial housing exposure (owners outright, owners with a mortgage, and private renters). Those reporting “other” as their tenure type were excluded from the analysis.

#### Covariates

**Figure 1.**
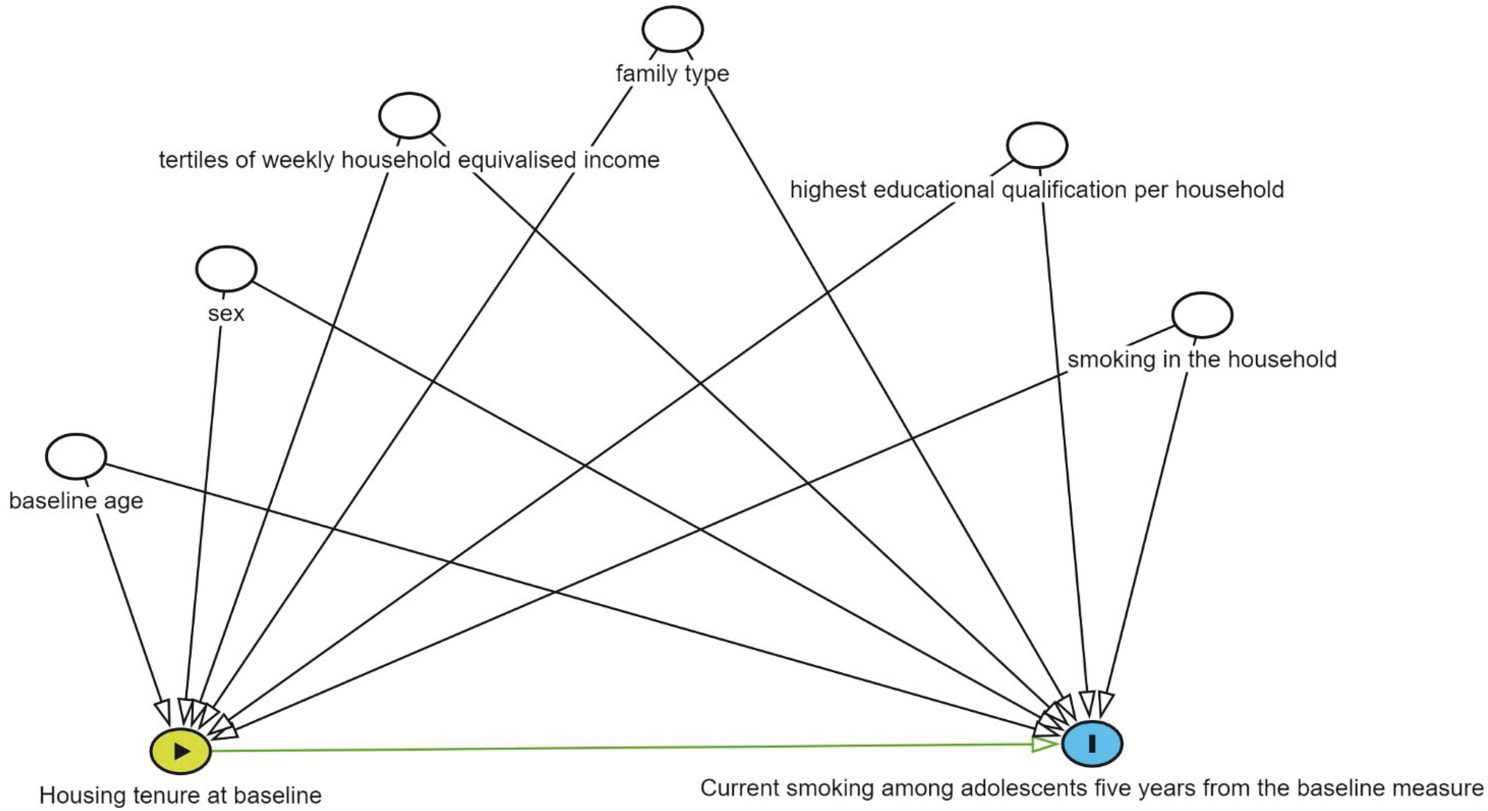
Directed acyclic graph for the association between housing tenure at baseline and current smoking among adolescents 5 years from the baseline measure of their housing and smoking status.

Multiple indicators of disadvantage are associated with housing tenure and high smoking prevalence.^11^ We accounted for baseline age (15- to 18-year-old), sex (male/female), tertiles of weekly household equivalized income, family type (couples/others, lone person, nonfamily member/lone parent), highest educational qualification per household (less than secondary/diploma, certificate, secondary/more than equal to a Bachelor degree), and smoking in the household. Following the disjunctive cause criterion, we adjusted for variables that are causes of exposure, or outcome, or both discarding any instrumental variable.^24^ The theorized relationships between the exposure, outcome, and confounders are presented in the directed acyclic graph (see Figure 1).

#### Statistical Analysis

Descriptive analysis was conducted to summarize the sample characteristics and map the distribution of covariates by housing tenure. First, we examined trends over time by plotting the prevalence of smoking by tenure among adolescents cross-sectionally. Next, we applied an inverse probability weighting approach to estimate the effect of social housing tenure on risk of smoking 5 years later. The inverse probability weighting approach maximizes exchangeability between the exposed and unexposed groups by assigning weights to balance the identified confounding factors in the exposed and unexposed groups. Therefore, after weights are applied, the differences between those in social housing and other tenures in terms of covariates (age, sex, household equivalized income, highest qualification in household, and household smoking) are minimized in the pseudopopulation.^25,26^ Once those in social housing and other tenures are now exchangeable, average probability of being a current smoker 5 years later across all participants is estimated by first assigning social housing to everyone and then counterfactually to other housing tenures. The difference in average probabilities generates the risk difference. The log of the ratio of averaged probabilities is exponentiated to generate risk ratios. We performed complete case analyses in Stata/MP 16.0.

## Results

Across the cohort of 15- to 18-year-old respondents, most lived in owner-occupied homes or in private rental (95%). Less than 5% resided in social housing (see Table 1). Adolescents living in social housing were in households that were socioeconomically less well off than their counterparts in other tenures in terms of household income and educational attainment. Almost half were from households with no adult who had completed secondary school, lone-parent households, and households in the bottom tertile of equivalized household income (Table 1). Smoking at baseline was patterned by tenure. Nearly one-quarter (23.3%) of adolescents reported being smokers by their early 20s. This was considerably higher among those in social housing (39.8%), followed by privately rented households (26.7%) and owner-occupied (18%). Within the social housing cohort, 62% lived with a smoker which was also much higher than for other tenures (see Table 1).

**Table 1.** Descriptive Characteristics of the Sample (*n* = 3132)

	Outright owner	Mortgage	Private renter	Public renter (social housing)
	%	%	%	%
Age at study entry				
15	76.6	84.5	79.9	74.7
16	9.2	6.5	10.0	12.3
17	7.2	4.9	6.1	6.6
18	6.9	4.1	4.0	6.4
Equivalized income (in quantiles)				
1 (lowest)	28.6	22.3	36.0	76.2
2 (medium)	32.0	35.6	42.4	21.5
3 (highest)	39.5	42.1	21.6	2.4
Sex				
Male	48.4	48.8	48.2	51.0
Female	51.7	51.2	51.9	49.0
Maximum household education				
Less than secondary	7.9	9.3	22.1	44.0
Diploma, certificate, or year 12	47.5	51.5	56.3	46.2
Bachelor or above	44.6	39.2	21.6	9.8
Household structure				
Coupled	89.6	89.2	62.4	51.3
Lone parent	10.4	10.8	37.6	48.7
Other	0.0	0.1	0.1	0.0
Household smoking				
No	79.7	73.9	52.0	37.9
Yes	20.3	26.2	48.0	62.1
Smoking status at baseline				
Do not smoke	95.2	94.6	88.3	86.6
Smoke	4.8	5.4	11.7	13.4
Smoker after 5 years				
Do not smoke	82.5	82.0	73.4	60.2
Smoke	17.5	18.0	26.7	39.8

**Figure 2.**
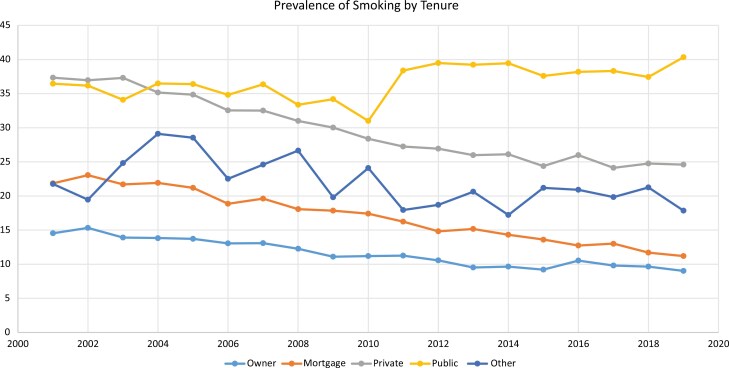
Prevalence of current smoking by housing tenure over time for 15–18 years (*N* = 3132).

Except for adolescents in public housing, the prevalence of smoking in other housing tenures either did not substantially change or reduced. The prevalence of smoking for adolescents in social housing increased by about 5% between 2000 and 2019 (see Figure 2).

Adolescents in social housing had 17% greater risk (95% confidence interval [CI] 0.02 to 0.31) of smoking uptake 5 years later when compared to those living in other tenures. On a relative scale, adolescents in social housing had 1.8 times higher risk (95% CI 0.95 to 2.66) of smoking uptake within 5 years than those in other tenures (see Table 2).

**Table 2.** Effect of Social Housing Tenure on Current Smoking 5 Years Later (*n* = 2727)

Tenure type	Risk difference (95% CI)	Risk ratio (95% CI)
Owner/private rental	REF	REF
Social housing	0.165 (0.02 to 0.31)	1.80 (0.95 to 2.66)

CI = confidence interval.

## Discussion

Our results confirm that adolescents in social housing have a higher risk of smoking initiation than comparable cohorts in other tenures—contributing to ongoing social disparities in smoking rates in Australia. The high smoking prevalence among young people in social housing persists over time, despite an overall decrease in adolescent smoking rates in Australia and despite several states and territories banning smoking in shared areas of multiunit public housing.^27^ This is a concerning finding, revealing structural and social inequalities in the gains that have been made in reducing smoking rates in high-income countries.

One plausible explanation for our findings of the differential rates of uptake between people in other tenures and social rental sectors, similar to as previously documented in the United States,^12^ is the low social housing provision in Australia. Low stock of social housing creates communities of disadvantage. As the demand for this low stock is extremely high, people in greatest need are prioritized. Due to the well-established role of social context in smoking,^7^ it is possible that the causal effect of social housing on smoking uptake may appear to be high primarily due to concentration of disadvantage, as there is no other mechanism through which social housing may lead to higher smoking rates. It is necessary to shift the attention to upstream interventions such as increasing social housing stock so that social housing does not reflect poverty but rather large-scale housing assistance for general population, for example, in Singapore. Such an approach will also minimize potential stigmatization of people living in social housing that is possible due to targeting low-income households or social housing residents with bans and/or education campaigns.

Moreover, the use of smoking bans in common areas in public housing^28^ has not curbed trends in smoking over time, reinforcing a focus on housing people as well as a tobacco reduction strategy. Ensuring social mixing of tenures and income groups in affordable housing developments to reduce revisualization and implementing models of governance of social housing that promote social capital in communities^29,30^ should be tested as prevention strategies in relation to smoking trends over time. While social housing might underwrite a “geography of harm,”^31^ it may well offer opportunities for a geography of harm minimization.^32,33^

Our study has important strengths. A robust method applied to a large cohort of adolescents from a comprehensive, national longitudinal study gave us the opportunity to examine how the relationship between social conditions and tobacco use plays out over time. Nonetheless, there are some limitations to our study. First, we relied on self-reported smoking. Smoking status is prone to measurement error with some young people in the HILDA study likely to underreport their tobacco use. It is unknown if the underreporting of tobacco use is likely to vary according to social disadvantage, therefore, our estimates can be biased in either direction (toward or away from null). Second, there is attrition dependent on gender, age, country of birth, socioeconomic status, and area of residence in HILDA sample over time. As adolescents experiencing social disadvantage are more likely to drop out, the smoking prevalence 5 years later is likely to be higher in the missing group. Therefore, our effect estimates are biased toward null due to potential selection bias.

## Conclusions

Public health and medical research have generated unequivocal evidence that smoking tobacco is bad for people’s health in the short, medium, and long term. Tobacco use exacerbates poverty and can have critical health and well-being implications across the life course.^34,35^ While our research points to social housing tenure as a residential context that increases the likelihood of smoking initiation in Australia, the challenge is to better understand why this is the case so as to design and offer social housing settings that are healthy for residents.^36,37^ This study extends our health knowledge of the potential pathways through which aspects of the social environment intersect with home environments and may affect tobacco use in adolescents. Policies aimed at reducing smoking in adolescents may need to go beyond the “nudge” strategies^38^ typical of an individualized harm reduction approach,^39^ to better support social inclusion, collective efficacy, and informal social interventions.^40^

## Data Availability

This paper uses unit record data from HILDA survey conducted by the Australian Government Department of Social Services (DSS). The findings and views reported in this paper, however, are those of the author(s) and should not be attributed to the Australian Government, DSS, or any of DSS’ contractors or partners (doi:10.26193/3QRFMZ).
